# Population tobacco control interventions and their effects on social inequalities in smoking: systematic review

**DOI:** 10.1136/tc.2007.023911

**Published:** 2008-05-19

**Authors:** S Thomas, D Fayter, K Misso, D Ogilvie, M Petticrew, A Sowden, M Whitehead, G Worthy

**Affiliations:** 1 MRC Social and Public Health Sciences Unit, Glasgow G12 8RZ, UK; 2 Centre for Reviews and Dissemination, University of York, York YO10 5DD, UK; 3 PEHRU, London School of Hygiene and Tropical Medicine, London WC1E 7HT, UK; 4 Division of Public Health, University of Liverpool L69 3GB, UK; 5 MRC Epidemiology Unit, Cambridge CB2 0QQ, UK

## Abstract

**Objective:**

To assess the effects of population tobacco control interventions on social inequalities in smoking.

**Data sources:**

Medical, nursing, psychological, social science and grey literature databases, bibliographies, hand-searches and contact with authors.

**Study selection:**

Studies were included (n = 84) if they reported the effects of any population-level tobacco control intervention on smoking behaviour or attitudes in individuals or groups with different demographic or socioeconomic characteristics.

**Data extraction:**

Data extraction and quality assessment for each study were conducted by one reviewer and checked by a second.

**Data synthesis:**

Data were synthesised using graphical (“harvest plot”) and narrative methods. No strong evidence of differential effects was found for smoking restrictions in workplaces and public places, although those in higher occupational groups may be more likely to change their attitudes or behaviour. Smoking restrictions in schools may be more effective in girls. Restrictions on sales to minors may be more effective in girls and younger children. Increasing the price of tobacco products may be more effective in reducing smoking among lower-income adults and those in manual occupations, although there was also some evidence to suggest that adults with higher levels of education may be more price-sensitive. Young people aged under 25 are also affected by price increases, with some evidence that boys and non-white young people may be more sensitive to price.

**Conclusions:**

Population-level tobacco control interventions have the potential to benefit more disadvantaged groups and thereby contribute to reducing health inequalities.

Reducing social inequalities in health is a priority for health policy in many countries.^1^ Although the extent and causes of health inequalities have been extensively researched, we know remarkably little about the actual effects of measures to reduce such inequalities,^2^ and it is possible that a strategy that improved health in the population overall might actually widen inequalities between social groups if its benefits were concentrated among the better-off.^3^

Smoking has been shown to be a major contributor to social inequalities in mortality and is the single greatest contributor to preventable illness and premature death in the United Kingdom.^4^ ^5^ The importance of interventions to reduce the association of smoking with disadvantage is well recognised^6^ and is reflected, for example, in the target set by the Department of Health to reduce the prevalence of smoking in “manual groups” from 32% to 26% by 2015.^7^ Smokers from lower socioeconomic groups may be less likely than those from higher socioeconomic groups to quit as a result of participating in individually targeted approaches such as smoking cessation services, although this social gradient in quit rates may be offset by a greater penetration of smoking cessation services in disadvantaged areas.^8^ The potential contribution of population-level interventions, such as restrictions on tobacco advertising and on smoking in public places, to reducing social inequalities in smoking has been less well researched.^9^ We carried out a systematic review of the differential effects of population-level tobacco control interventions by evaluating their effects in groups with different demographic and socioeconomic characteristics. Our overall aim was to identify which interventions are most likely to be effective in reducing smoking-related health inequalities.

## METHODS

### Search strategy

We identified primary studies in any language by searching medical, nursing, psychological, social science and grey literature databases from their inception dates to January 2006. We did not limit our searches by study design. We also examined bibliographies and conference abstracts, hand-searched key journals and contacted authors for additional information where necessary. Further details can be found in our full report at http://www.york.ac.uk/inst/crd/projects/tobacco-control.htm.

### Study selection and inclusion criteria

Titles and abstracts were assessed for relevance independently by two reviewers. Potentially relevant studies were assessed for inclusion independently by two reviewers, with disagreements resolved through discussion and, where necessary, the involvement of a third reviewer.

We included studies of any design that assessed the effects of a population-level tobacco control intervention (see box) in smokers, people at risk of taking up smoking, people at risk of exposure to environmental tobacco smoke (ETS) or the general population. Studies had to report quantitative outcomes for individuals or groups with different demographic or socioeconomic characteristics. Eligible outcomes included changes in smoking behaviour (such as prevalence or consumption), indirect measures of tobacco consumption (such as illegal sales to minors or quantity of smuggled cigarettes), exposure to ETS, intermediate outcomes (such as changes in knowledge or attitudes), process measures (such as participation rates), implementation measures (such as enforcement of policy changes) and any health outcomes (such as mental health or wellbeing), as well as adverse or unintended effects. We also included qualitative data where these were linked to an included quantitative study. We excluded studies of interventions conducted exclusively within closed settings (such as psychiatric or addiction treatment facilities, detention centres or prisons) because this review was concerned with effects in the wider population. We also excluded studies that assessed the effects of restrictions on sales to minors (youths) by only reporting test purchases as outcomes. This is because we considered the minors undertaking the test purchases at retail outlets to be part of the intervention, their purchase attempts being a device for evaluating the implementation and enforcement of the intervention. Such “test purchases” alone did not provide sufficient data for our purposes on the differential effects of an intervention between social groups. We did, however, include studies that assessed the effects of restrictions on sales to minors by reporting evaluation data from a larger population (such as surveys of local schoolchildren).

**What is a population-level tobacco control intervention?**

We defined population-level tobacco control interventions as those applied to populations, groups, areas, jurisdictions or institutions with the aim of changing the social, physical, economic or legislative environments to make them less conducive to smoking. These are approaches that mainly rely on state or institutional control, either of a link in the supply chain or of smokers’ behaviour in the presence of others. Our definition was based on our pilot study^10^ and scoping searches for the systematic review and includes interventions such as:

- Tobacco crop substitution or diversification
- Removing subsidies on tobacco production
- Restricting trade in tobacco products
- Measures to prevent smuggling
- Measures to reduce illicit cross-border shopping
- Restricting advertising of tobacco products
- (Enforcing) restrictions on selling tobacco products to minors
- Mandatory health warning labels on tobacco products
- Increasing the price of tobacco products
- Restricting access to cigarette vending machines
- Restricting smoking in the workplace
- Restricting smoking in public places.

Such approaches could also form part of wider, multifaceted interventions in schools, workplaces or communities.

We did not include interventions whose main aim was to strengthen the capacity of individuals to stop smoking or to resist taking up smoking, even if these interventions were applied to whole groups or populations (for example, mass media health education campaigns). These are approaches that mainly rely on individuals engaging voluntarily with measures intended to help them.

### Data extraction and quality assessment

Data were extracted and the quality of each study was assessed independently by one reviewer and checked by a second. We summarised study quality using a scale of suitability of study design adapted from the criteria used for the Community Guide of the US Task Force on Community Preventive Services^11^ and a six-item checklist of quality of execution adapted from the criteria developed for the Effective Public Health Practice Project in Hamilton, Ontario^12^ (see table on *Tobacco Control* website). We extracted outcome, process and implementation data stratified by the sociodemographic characteristics specified in the PROGRESS criteria (place of residence, race or ethnicity, occupation, gender, religion, educational level, socioeconomic status (for example, represented by income), and social capital)^13^ and also by age for interventions targeted at populations considered specifically “at risk” of smoking because of their age (adolescents and young adults). For studies where it appeared that relevant data on differential effects may have been collected but not reported, we contacted authors to request additional data.

Data from qualitative studies were extracted using methods adapted from those developed by Britten *et al*^98^ and their quality was assessed using published prompts for appraising qualitative research.^99^ Any disagreements at each stage were resolved by discussion and, if necessary, the involvement of a third member of the review team.

### Data synthesis

**Figure 2:**
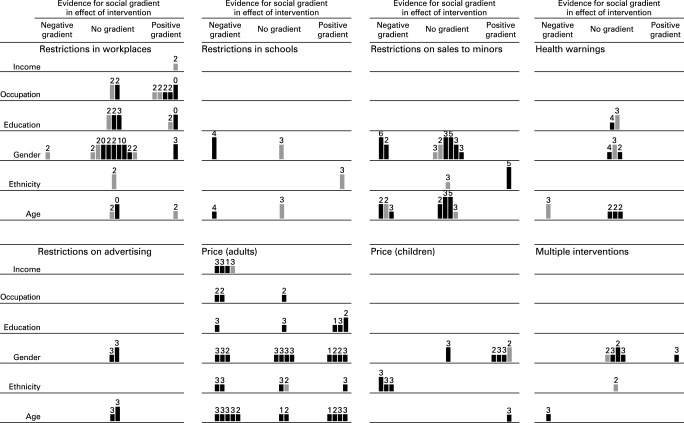
Evidence for social gradients in effects of interventions. A „supermatrix” covering all categories of intervention consisting of six rows (one for each dimension of inequality) and three columns (one for each of the three competing hypotheses about the differential effects of each category of intervention). Each study is represented by a mark in each row for which that study had reported relevant results. Studies with hard behavioural outcome measures are indicated with full-tone (black) bars, and studies with intermediate outcome measures with half-tone (grey) bars. The suitability of study design is indicated by the height of the bar, where the highest bars represent the most suitable study designs (categories A and B) and the lowest bars represent the least suitable (category D). Each bar is annotated with the number of other methodological criteria (maximum six) met by that study.

We adopted a hypothesis-testing approach to examine the balance of evidence about the differential effects of interventions and synthesised the data using a combination of graphical and narrative methods, including a novel matrix or “harvest plot” (see fig 2).^100^ For each category of intervention and dimension of inequality, we populated the relevant row of this matrix by placing a bar representing each study in one of three columns according to which of three competing hypotheses were most strongly supported by the results of that study:

- The null hypothesis that for any given demographic or socioeconomic characteristic there was no social gradient in the effectiveness of the intervention
- The alternative hypothesis that there was a positive social gradient in effectiveness, meaning that the intervention was more effective in more advantaged groups (defined for this purpose as the more affluent, those with a higher level of education, those in more skilled occupational groups, males, older people or those in the majority or most advantaged racial or ethnic group in the context of a particular study)
- The alternative hypothesis that there was a negative social gradient in effectiveness, meaning that the intervention was more effective in more disadvantaged groups.

## RESULTS

**Figure 1:**
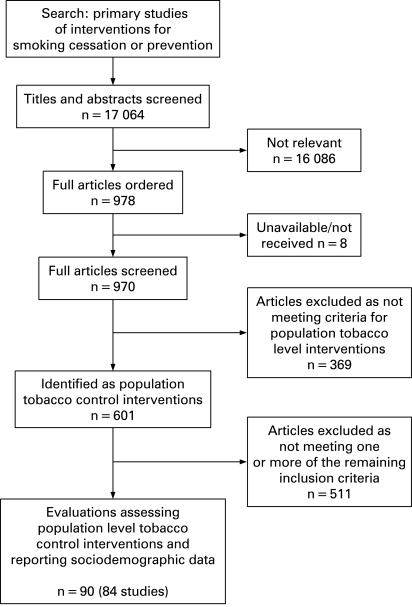
Process of study selection.

We screened a total of 17 064 references, identified 970 potentially eligible papers and finally included 84 studies (reported in 90 papers) (fig 1). We found only one qualitative study conducted in conjunction with a quantitative study.^22^ We approached six authors for additional data but none was forthcoming.

We found relevant evidence for seven categories of intervention: restrictions on smoking in workplaces and public places, restrictions on smoking in schools, restrictions on sales to minors, health warnings on tobacco products, restrictions on advertising of tobacco products, price of tobacco products and multifaceted interventions (see fig 2). Further details of the studies included in each category can be found in our full report at http://www.york.ac.uk/inst/crd/projects/tobacco-control.htm.

The included studies reported outcomes by race or ethnicity, occupation, gender, educational level, income or age. As no studies reported outcomes by place of residence, religion or level of social capital these characteristics were excluded from our analysis.

Stronger designs tended to have been used for studies of the effects of restrictions on smoking in workplaces, public places and schools and restrictions on sales to minors, of which three were cluster randomised controlled trials.^31^ ^32^ ^34^ Studies of other types of intervention were predominantly cross-sectional or retrospective.

Studies of restrictions on sales to minors were the most likely to fulfil the criteria for quality of execution, with one study meeting all six criteria^31^ and two studies meeting five.^32^ ^34^ Two studies of restrictions on smoking in schools met four criteria.^28^ ^29^ The remaining studies in this review met between zero and three of the criteria.

### Restrictions on smoking in workplaces and public places

Fourteen studies, nine published between 1981 and 1999 and five published more recently, evaluated smoking restrictions or bans in the workplace or in public places^14^^–^27 in the United States,^14^ ^16^ ^20^ ^21^ ^23^^–^26 Australia,^15^ New Zealand,^27^ Israel,^17^ Finland,^18^ Scotland^22^ and Wales.^19^ The interventions consisted of a total ban on indoor smoking,^14^ ^15^ ^17^ ^24^ ^25^ ^27^ a smoking ban with exceptions,^22^ restricting smoking to designated rooms or areas^18^ ^19^ ^21^ ^23^ or displaying no-smoking signs in a hospital lobby.^16^ The nature of the smoking ban was unclear in two studies.^20^ ^26^ The balance of evidence from five comparatively weak studies suggested that, if anything, restrictions on smoking in workplaces may be more effective for staff in higher occupational grades.^19^ ^22^^–^25 We found insufficient evidence of differential effects by income,^26^ educational level^14^ ^17^ ^18^ ^25^ ^26^ or ethnicity,^27^ inconsistent evidence of differential effects by age, and no evidence of differential effects by gender.^14^^–^21 24^–^26

### Restrictions on smoking in schools

Three studies assessed the effects of restrictions on smoking in schools, one published in 1999^29^ and two published in 2005.^28^ ^30^ These examined the effects of a smoking policy in a UK school,^29^ student beliefs and support for a school smoking ban in a mostly non-white population in California^30^ and the effects of enforcement action on student smoking behaviour and attitudes in another US population.^28^ These studies suggested that restrictions on smoking in schools may be more effective in girls than in boys^29^ and in middle-school than in high-school students,^28^ and that attitudes were more favourable in non-Hispanic students than in Hispanic students.^30^ No studies provided evidence about possible differential effects by parental income, occupation or educational level.

### Restrictions on sales to minors

Thirteen studies, most published between 2000 and 2005, evaluated restrictions on sales to minors in the United States,^31^^–^34 36 38 42 Sweden,^41^ Finland,^37^ Australia^39^ ^40^ ^43^ and New Zealand^35^ in populations aged between 13 and 18 years of age. The interventions included education of retailers and the community, enforcement of legislation, or both. The evidence from two studies (one of an educational intervention and one of combined education and enforcement) suggested that girls may be less likely to use tobacco as a result of the intervention than boys.^31^ ^33^ The evidence from six other studies (four of an enforcement intervention and two of combined education and enforcement) on differential effects by gender was inconsistent.^32^ ^35^ ^37^ ^39^^–^41 One study of combined education and enforcement found that the intervention was less effective in non-white students than in white students.^34^ A second weaker study of an enforcement intervention found no evidence of differential effects by ethnicity.^35^ Three studies (two of an enforcement intervention and one of combined education and enforcement) found larger effects in younger students than in older students.^33^ ^37^ ^41^ Four other studies (one of an enforcement intervention and three of combined education and enforcement) found inconsistencies in effects by age.^32^ ^35^ ^39^ ^43^ No studies provided evidence about possible differential effects by parental income, occupation or educational level.

### Health warnings on tobacco products

Five studies assessed the effects of health warnings and labelling of contents on tobacco products in the general population,^46^ ^47^ ^50^ young adults^48^ or schoolchildren.^49^ Studies were published between 1997 and 2005 and were conducted in Australia,^46^ Canada,^47^ ^48^ the United States^49^ and The Netherlands.^50^ We found no consistent evidence of differential effects on smoking behaviour by education for smoking behaviour^46^ ^50^ or on smoking attitudes or behaviour by gender.^46^ ^48^ ^50^ In three studies of young people, health warnings did not appear to change attitudes or smoking behaviour.^47^^–^49 No studies provided evidence about possible differential effects by income, occupation or ethnicity.

### Restrictions on advertising of tobacco products

Two studies assessed the effects of advertising restrictions on children and young people. One study was set in Hong Kong and published in 2004.^44^ The other used national statistics from 1992 to assess smoking prevalence among adolescents in Norway, Finland, New Zealand and France.^45^ We found no evidence of differential effects by gender or age. No studies provided evidence about possible differential effects by parental income, occupation, educational level or ethnicity.

### Price of tobacco products

Forty-two studies provided information about the effects of the price of tobacco products on smoking behaviour. Most were econometric analyses applying statistical models to cross-sectional or longitudinal survey data from various time periods between 1961 and 2003. These studies modelled the relation between the decision to smoke or the quantity of cigarettes smoked and changes in price or tax. Most used survey data from the United States with 20 studies reporting data for adolescents or college students only^52^ ^56^ ^57^ ^60^ ^61^ ^64^ ^68^ ^69^ ^72^ ^76^ ^78^^–^83 88 89 91 92 and 13 reporting data for adults only or for young people and adults combined.^54^ ^55^ ^58^ ^59^ ^62^ ^63^ ^65^^–^67 71 74 77 87 Three studies were conducted in the United Kingdom^53^ ^84^ ^85^ while others were from France,^75^ Spain,^73^ Canada,^90^ South Africa^51^ and Taiwan.^70^ ^86^

### Effects on adults

Four studies found that cigarette price increases had a greater effect in those on lower incomes.^59^ ^66^ ^70^ ^90^ Two UK studies found that effects on smoking were greater among those in manual occupations than those in professional occupations^84^ ^85^ but a later UK study found no evidence of differential effects by occupation.^53^ There was also some evidence to suggest that those with higher levels of education may be more sensitive to price.^70^ ^77^ ^86^ We found no clear evidence for differential effects by gender or ethnicity.

### Effects on young people

All 20 studies restricted to adolescents or college students found that these groups were sensitive to price and concluded that increasing the price of tobacco products would reduce youth smoking.^52^ ^56^ ^57^ ^60^ ^61^ ^64^ ^68^ ^69^ ^72^ ^76^ ^78^^–^83 88 89 91 92 The only study comparing children within different age groups found that those aged 17 or 18-years-old were more sensitive to price increases than those aged between 13 and 16-years-old.^68^ Four studies found that boys aged 13–18 were more sensitive to price than girls.^76^ ^88^ ^89^ ^91^ All three studies which examined effects by ethnicity found that black or Hispanic adolescents were more affected by price increases than their white counterparts.^68^ ^88^ ^92^ No studies provided evidence about possible differential effects by parental income, occupation or educational level.

### Multifaceted interventions

Five studies assessed the effects of combinations of interventions, mainly the combined effects of different anti-tobacco laws.^93^^–^97 Studies were published between 1997 and 2004. Two studies examined the impact of the 1976 National Tobacco Control Act in Finland.^94^ ^95^ One study assessed the impact of French legislation including restrictions on smoking in the workplace, advertising restrictions, health warnings on tobacco products and restrictions on sales to minors. This study involved a survey of hospital employees, mainly female nurses and healthcare workers.^93^ One study assessed smoking restrictions in Californian schools as part of an independent evaluation of the Californian Tobacco Control Prevention and Education Program.^97^ The fifth study assessed the effects of price increases and tobacco control legislation in Canada.^96^ The effects of the components of these interventions were not assessed separately within the studies and we therefore classified them as multifaceted interventions in our analysis.

One study found that the introduction of a tobacco control act in Finland reduced the rate of smoking initiation among young people.^94^ We found no evidence of differential effects by gender (interventions in all four studies were effective for both men and women)^93^^–^95 97 or ethnicity (one study).^97^ No studies provided evidence about possible differential effects by income, occupation or educational level.

## DISCUSSION

### Principal findings

This review has systematically and comprehensively applied an “equity lens” to tobacco control interventions, re-examining the available evidence about the impact of policy measures and other population-level interventions in order to assess their role in tackling health inequalities.^101^

The literature is international, with over half of the studies having been conducted in the United States and just six in the United Kingdom, and is dominated by econometric analyses (half of the included studies) modelling the effects of the prices of tobacco products.

Overall, we found no strong evidence that restrictions in workplaces and public places are more effective in reducing smoking in more advantaged groups, although smoking behaviour and attitudes may be more favourably affected among those in higher occupational grades.

We found evidence from single studies that smoking restrictions in schools may be more effective in girls and in younger schoolchildren, but there was an absence of evidence with respect to other possible differential effects. We found more, better-quality evidence on the differential effects of restrictions on sales to minors: restrictions seem to be more effective in girls and in younger schoolchildren, and one study of a combined education and enforcement intervention found restrictions on sales to minors to be more effective in white than non-white groups. For health warnings on tobacco products and restrictions on tobacco advertising, the lack of robust studies makes firm conclusions difficult. The effects of health warnings do not appear to be subject to a sociodemographic gradient, but their effects have not been examined with respect to income, occupation or ethnicity and the evidence with respect to educational level, gender and age is not convincing. The effects of advertising bans also show no differential by gender or age, but the evidence is not strong and other potential gradients have not been examined in primary studies.

The balance of econometric evidence suggests that increasing the price of tobacco is more effective in reducing smoking in lower-income adults and those in manual occupations. There was also some evidence to suggest that smokers with higher levels of education may be more responsive to price, although this evidence was limited to somewhat specific study populations (men in Taiwan and pregnant women in the United States, whose response to pricing may be confounded by knowledge of the risks of smoking during pregnancy). The evidence with respect to differential effects by gender, ethnicity or age is not consistent. Although we found fewer studies assessing the effects of pricing in children, it appears that boys, non-white children and perhaps also older children may be more price-sensitive. We found no evidence as to how the effects on children varied by household income.

### Strengths and weaknesses of the review

We made extensive attempts to obtain both published and unpublished studies and to include a wide range of study designs in order to avoid overlooking evidence from weaker studies which to date have mainly been excluded from systematic reviews. However, it remains possible that we have not identified all relevant tobacco control intervention programmes or policies, given that some may not have been formally evaluated or reported.

One difficulty in dealing with a diverse public health evidence base is the need to incorporate considerable heterogeneity in intervention, study design and appropriateness of that design, study quality and study outcomes (in this case, “hard” behavioural and “softer” attitudinal outcomes). The stratification of outcomes by social group adds another level of complexity. To manage this we developed a novel graphical method, the “harvest plot”, to synthesise and display the balance of evidence to support competing hypotheses about possible social gradients in the effects of the interventions. This methodological development is a considerable strength of the review and may be of use to others reviewing the public health literature; the rationale for this method and its advantages and disadvantages are discussed in a separate methodological paper.^100^

### Strengths and weaknesses of the available evidence

There are undoubted limitations in the evidence base, most notably a lack of prospective evaluations. A particular challenge is the difficulty of attributing outcomes solely to the intervention in question. Authors often did not report co-interventions or describe other contextual factors that might have influenced the success of the intervention. Although we excluded studies focusing solely on individual-level interventions, population tobacco control policies rarely exist in isolation and several studies included individual-level interventions such as smoking cessation classes alongside workplace smoking bans. A decision to intervene at one level (policy) could be adversely affected by actions at other levels; alternatively, there could be a synergistic effect.^102^ Contextual information would also help policy-makers and practitioners better understand how successful interventions could be implemented.^103^

The completeness and clarity of reporting in primary studies in this field would also be improved by the inclusion of more methodological details (such as study design, sampling, population characteristics, data collection tools, methods of analysis and attrition rates), by assessing the differential impact of interventions across different sociodemographic groups and by reporting data on changes in smoking behaviour rather than relying on changes in attitudes which may be a poor proxy for behaviour change. One of the more obvious limitations is the absence of qualitative research on population-level tobacco interventions and their effects on social inequalities in smoking. Although we sought such studies, we found only one. New qualitative research will also have an important part to play in identifying intended and unintended effects of policy interventions and barriers to change before implementation.^102^

### Implications for policy and practice

The current EU green paper on policy options for progressing towards a “smoke-free Europe” notes that smoke-free policies may reduce socioeconomic inequalities in health and calls for qualitative and quantitative evidence on the impacts of such policies.^104^ Our systematic review addresses this call, contributes a step towards understanding the interventions that are effective for different social groups and may inform decisions about tackling social inequalities in smoking.

The most compelling evidence of a social gradient in effectiveness which favours the least well off is for the price of tobacco products; although we also found some evidence to suggest an apparently greater effect of price on those with higher levels of education, such evidence is limited and requires further investigation. Increasing the price of tobacco is therefore the population-level intervention for which we found the strongest evidence as a measure for reducing smoking-related inequalities in health. However, the effects of increasing tobacco taxation may be undermined by tax-evasion or tax-avoidance measures such as smuggling and cross-border shopping.^105^ The Acheson inquiry^106^ and other commentators^107^ ^108^ have also raised concern about the long-term effect of price rises on disadvantaged households, where smokers are more likely to be nicotine-dependent and for whom living in hardship is the primary deterrent to quitting. Any further increase in tobacco taxation would therefore require extra measures to support cessation among low-income households.

None the less, we found more evidence to support increasing the price of tobacco products than to support other more visible interventions such as health warnings and advertising restrictions, whose differential effects appear under-explored. However, although interventions such as health warnings and advertising restrictions may not in themselves affect inequalities, they may be important as part of a wider tobacco control strategy, if they help to elicit public support for other measures.^109^

The evidence on restrictions on sales to minors suggests that these may be effective in deterring younger smokers, though their effectiveness depends on enforcement as unenforced voluntary agreements with retailers are less effective in reducing sales.^105^ Pricing may be less effective among some groups of younger smokers, perhaps because they may obtain their cigarettes from non-commercial sources.^105^ Among younger smokers restrictions in schools (which affect consumption) and health warnings (which affect attitudes to smoking) may therefore be more productive. Appropriately enforced restrictions on sales to minors may offer the greatest promise as part of a strategy for tackling inequalities. While combinations of interventions are also likely to be an important part of the policy armoury—including restrictions in schools (which affect consumption) and health warnings (which affect attitudes to smoking)—the differential effects of such combinations largely remain an area for further research.

It is also important to identify policies that have the potential to *increase* inequalities. Our findings are encouraging, as we found little evidence of adverse effects in this regard. One exception was workplace restrictions, which may be more effective among higher occupational grades. This suggests that the implementation of such policies should be accompanied by measures to promote adherence across all occupational grades. This supports the case for legislating for mandatory workplace bans, rather than relying on willing employers to introduce voluntary bans.

### Unanswered questions and future research

We have identified many gaps in the evidence base on interventions to reduce social inequalities in smoking. In particular, we know little about the differential effects of most categories of intervention by income, gender or ethnicity. For tobacco pricing—a relatively well researched field—we also need to know more about effects on adolescents from lower-income households and on young people in general, and on lower-income adults who are likely to be nicotine-dependent. For restrictions on sales to minors—another relatively well researched field—it is unclear whether differential effects vary between interventions that involve education, enforcement or both. Where population-level studies are carried out there could be greater use of pre-planned subgroup analyses, specifically to shed light on effects on inequalities, but there also remains a need for robust evaluations of targeted interventions (even accepting that these may not provide evidence about effects on inequalities). Perhaps the most important observation is that much of the existing evidence derives from the United States. The greatest research priority should therefore be to develop relevant evidence for other country contexts with a focus on behavioural outcomes. The introduction of new population-level tobacco control policies—such as the restrictions on smoking in public places now introduced in all the countries of the United Kingdom and elsewhere—provides such an opportunity.

**What is already known on this subject**

- Reducing social inequalities in smoking and its health consequences is a public-health and political priority.
- Little is known about the actual effects of measures to reduce health inequalities in general or about the differential impacts of tobacco control measures in particular.
- It is possible that a strategy which successfully reduced smoking in the population overall might widen inequalities if its benefits were concentrated among the better-off.

**What this study adds**

- This is the most comprehensive review to date of the potential effects on heath inequalities of population-level tobacco control interventions and makes an important contribution towards understanding the effects of interventions in different social groups.
- In terms of reducing social inequalities in smoking, we found better evidence to support increasing the price of tobacco products than to support more visible interventions such as health warnings and advertising restrictions.
- We found little evidence of policies that have the potential to increase inequalities. In particular, we found no strong evidence that smoking restrictions in workplaces and public places are more effective among more advantaged groups.
